# Minimizing Hemodialysis Catheter Dysfunction: An Ounce of Prevention

**DOI:** 10.1155/2012/170857

**Published:** 2012-02-19

**Authors:** Timmy Lee, Charmaine Lok, Miguel Vazquez, Louise Moist, Ivan Maya, Michele Mokrzycki

**Affiliations:** ^1^University of Cincinnati, Cincinnati, OH 45267-0585, USA; ^2^Faculty of Medicine, University of Toronto, University Health Network-Toronto General Hospital, Toronto, ON, Canada M5G 2C4; ^3^University of Texas Southwestern Medical Center, Dallas, TX 75390-8856, USA; ^4^Department of Epidemiology and Biostatistics, University of Western Ontario, London, ON, Canada N6A 5W9; ^5^Division of Nephrology, Department of Medicine, University of Western Ontario, London, ON, Canada N6A 5W9; ^6^Nephrology Associates of Central Florida, Lake Mary, FL 32746, USA; ^7^Albert Einstein College of Medicine, Bronx, NY 10467, USA

## Abstract

The maintenance of tunneled catheter (TC) patency is critical for the provision of adequate hemodialysis in patients who are TC-dependent. TC dysfunction results in the need for costly and inconvenient interventions, and reduced quality of life. Since the introduction of TCs in the late 1980s, heparin catheter lock has been the standard prophylactic regimen for the prevention of TC dysfunction. More recently, alternative catheter locking agents have emerged, and in some cases have shown to be superior to heparin lock with respect to improving TC patency and reducing TC-associated infections. These include citrate, tissue plasminogen activator, and a novel agent containing sodium citrate, methylene blue, methylparaben, and propylparaben. In addition, prophylaxis using oral anticoagulants/antiplatelet agents, including warfarin, aspirin, ticlodipine, as well as the use of modified heparin-coated catheters have also been studied for the prevention of TC dysfunction with variable results. The use of oral anticoagulants and/or antiplatelet agents as primary or secondary prevention of TC dysfunction must be weighed against their potential adverse effects, and should be individualized for each patient.

## 1. Introduction

Tunneled catheters (TCs) are frequently used in patients who require both temporary and long-term hemodialysis (HD) but do not have a functioning arteriovenous fistula, graft, or peritoneal dialysis catheter [1]. About 20% of prevalent and 80% of incident HD patients in the United States use a TC and the proportion is even higher in some other countries [2, 3]. TCs have advantages and disadvantages as a vascular access for dialysis [1, 3, 4]. There is no need for a surgical procedure to place a TC or waiting for maturation prior to use. Thus, TCs are immediately available for use, and there are several different options on where to place them in most patients. Unfortunately, TCs have several major problems including frequent TC dysfunction and infections. Patients with TC have more hospitalizations, incur higher costs, and are at increased risk for inadequate dialysis and higher morbidity and mortality [1–6]. The main objective of this paper is to evaluate potential interventions to prevent HD TC dysfunction. According to the NKF Dialysis Outcomes Quality Initiative (KDOQI)-2006, dialysis access dysfunction is defined as an inability to achieve a dialysis blood flow rate of at least 300 mL/min during the first hour of dialysis despite at least one attempt to increase blood flow [1]. Several interventions including instillation of locking solutions and administration of systemic anticoagulation and antiplatelet agents are reviewed.  Table 1 is a summary of the most rigorously published clinical trials of interventions to prevent dialysis TC dysfunction [7–27].

## 2. TC Insertion

Several investigators have reviewed the principles of TC insertion [6, 28–30]. TC insertion should be performed with ultrasound guidance to identify the vein and guide the needle puncture [6, 31, 32]. The right internal jugular vein is the preferred site as it offers a more direct route to the right atrium and usually allows a smooth wide curvature to the TC [1, 6, 28, 29]. The use of fluoroscopy is strongly recommended to guide placement of the TC tip in the midatrium [1, 28]. TCs in the left internal jugular vein have a poorer blood flow than in the right internal jugular vein. TC in the subclavian vein can lead to subclavian vein stenosis and prevent future use of the ipsilateral upper extremity for a permanent vascular access [33]. Other sites such as femoral veins are not recommended as sites for TC insertion given the risks of infection and limitations to patient mobility.

## 3. The Role of Biofilm and Fibrin Sheath Formation in Hemodialysis Catheter Dysfunction

A biofilm is defined as a microbially derived, sessile community, characterized by cells that are attached to a substratum or to each other and are protected by a matrix of extracellular polymeric substances that they have produced [34]. One major complication of biofilms, in addition to infection, is the development of a fibrin sheath, which plays a major role in TC dysfunction.

While the pathophysiology of biofilm production is unclear, it has been hypothesized that biofilm formation occurs after initial contact of free-floating bacteria with a foreign surface, that is, dialysis TC, and can occur as early as 1–14 days after placement [35–40]. The bacteria initially attache and adhere irreversibly to the foreign surface, generate molecular signaling, and proliferate to transform into bacterial microcolonies [35–37]. Subsequently, the bacteria generate a coating of exopolysaccharide from the bacterial products so that their progeny can adhere firmly to the surface covered by a sticky glycocalyx matrix, called the biofilm, which envelopes the community of bacterial microcolonies [35–37]. Biofilms subsequently evolve and mature into a community of bacteria covered by a dense layer of matrix [35–37]. Furthermore, bacteria form biofilms preferentially in very-high-shear environments, similar to conditions that occur within the TC during the dialysis procedure, by enhancing bacterial adhesion [34]. Of note, the mere presence of a biofilm does not necessarily lead to infection and it may grow too slowly to produce clinical symptoms such as fever, chills and bacteremia. However, one important noninfectious complication of biofilm formation is the concurrent development of a fibrin sheath, which plays a major role in TC dysfunction.

Since the pathogenesis of biofilm development to fibrin sheath formation is not well understood, there is debate as to the sequence of fibrin sheath formation, biofilm development, and their interdependency [41]. However, the best evidence supports the hypothesis that a biofilm evolves over days to months into a more complex structure, a fibrin sheath. In addition to fibrin, these sheaths contain multiple other molecular and cellular components, including laminin, fibronectin, collagen, and smooth muscle cells, overlying endothelial cells [35]. These findings demonstrated that the TC-related fibrin sheath is an active response of the components of the vessel wall to the TC (e.g., biofilm formation) and associated thrombosis, as opposed to a mere deposition of acellular material and thrombus [42]. The fibrin sheath initiates at the point of contact between the TC and the vessel wall, advances along the entire length of the TC or device, and can create a one-way valve mechanism, with resultant decrease in the TC flow [42]. Fibrin sheaths play a major role in TC malfunction [35, 43–45]. Although the reported incidence of fibrin sheath can be as high as 100%, this condition may remain subclinical. However, in clinically affected (symptomatic) patients, the fibrin sheath may result in thrombus formation and malfunctioning TCs, in addition to infection [42, 43].

Once biofilms form, they are very resistant to antithrombotic and antimicrobial agents. There have been several recent clinical studies that have focused on investigating prophylactic pharmacologic treatment with antithrombotic and antimicrobial locking solutions and biological characteristics of bacterial TC adherence and biofilm formation showing some promising results [46, 47]. As with the paucity of therapies to prevent biofilm development, there are also few proven therapies to prevent fibrin sheath formation. The current treatment paradigms for management of fibrin sheaths in dysfunctional TCs include thrombolytic therapy, angioplasty with fibrin sheath disruption, and TC exchange [1, 42]. Transfemoral percutaneous fibrin sheath stripping is associated with poor patency. The best management strategy for TC malfunction is its prevention. The most common form of prevention is with the use of anticoagulant locking during the intradialytic period.

## 4. Heparin Catheter Lock

Heparin lock has been used for decades with relative safety; however, TC dysfunction continues to remain problematic. The rate of TC thrombosis associated with heparin lock in several large series ranges between 4–5.5 episodes/1000 days, and the rate of TC loss due to dysfunction is 1.8–3.6/1000 days [7–12]. The concentration of heparin used as a TC locking solution varies among studies, ranging between 1,000 U/mL and 10,000 U/mL. In a prospective trial comparing a period of high dose (10,000 U/mL) versus a period of low dose (1,000 U/mL) heparin lock, there was no significant difference in TC dysfunction or bleeding complications between heparin lock concentrations, although the need for TPA therapy was 4-fold higher (*P* < 0.001) when low-dose heparin lock was utilized [7]. Similar results were reported in a smaller, retrospective study comparing TPA administration in 2 HD units, one unit using high-dose heparin lock (10,000 U/mL) and the other using low-dose heparin lock (1,000 U/mL) [8]. Low-dose heparin lock was associated with 2-fold increase in TPA administration (*P* < 0.001). Theoretically, the use of high-concentration heparin lock may be advantageous in reducing both TPA-related costs and delay in treatment initiation; however, when Holley and Bailey took into account the differential cost/treatment between low- and high-dose heparin lock ($0.20 versus $2.67), low dose heparin resulted in significant savings despite higher TPA use [8]. Low-dose heparin lock (1000 U/mL) is particularly advantageous in the immediate postinsertion period [21]. In a retrospective analysis comparing heparin lock (5000 U/mL) versus heparin lock (1000 U/mL) or citrate administered immediately after TC insertion, patients receiving high-dose heparin lock (5000 U/mL) had a 9-fold increase in composite bleeding events (*P* = 0.01), and 7.7% experienced a major bleeding event (versus 0% in the low-dose heparin or citrate group) [48]. A significant increase in severe hemorrhage after TC insertion was associated with heparin lock (5,000 U/mL) in a large randomized controlled trial in comparison to citrate 30% lock, (rate of severe hemorrhagic events: heparin 13% versus citrate 4%, *P* = 0.005) [15].

The optimal volume of heparin lock should be individualized according to the patient and catheter characteristics. All dialysis TCs have some degree of locking solution leakage, depending on their design, even when <20% of the TC lock volume is instilled [49]. TC leak begins immediately after instillation and continues over a 30-minute period, and is higher in nontunneled catheters for both periods [50, 51]. The excess leakage volume is 0.16–0.48 mL with <20% TC fill volume, and 0.99–1.43 mL with >20% TC fill volume. TC lock overfill by 20% has been suggested to ensure delivery of heparin to the TC tip and wall and to improve TC patency which may be desirable in TCs with a history of recurrent thrombosis. Overfill by 20%, however, may result in the inadvertent systemic administration of substantial amounts of heparin and may be problematic in the pre-operative patient, or in patients with bleeding diatheses [49, 52]. Undesired systemic anticoagulation (increased activated partial thromboplastin time), caused by heparin lock overfill may persist for up to 4 hours [53]. The average rate of major bleeding episodes associated with heparin lock (5,000 U/mL) is approximately 2/1000 TC days [15].

Heparin lock has been traditionally administered in a thrice weekly dosing, with an interdialytic heparin dwell period. The results of a recently published small prospective study reported improved efficacy using a 6-day per week heparin locking regimen. This protocol is not practical in an outpatient HD setting, but may be useful in hospitalized patients with HD TCs. One caveat to this protocol, however, is that more frequent access of the TC lumen has the potential to increase the risk of infectious contamination if not using sterile procedures [54].

## 5. Trisodium Citrate Lock

In the last decade, trisodium citrate has emerged as an alternative to heparin as a TC locking solution. Seven clinical trials reported citrate lock (4%, 30%, or 46.7%) to be equivalent or superior to heparin lock (5,000–10,000 U/mL) with respect to the thrombolytic therapy rates and number of TCs removed for flow problems (Table 1 [6, 9–12]). In contrast, 2 studies reported unfavorable outcomes with citrate lock. The first was a small study (19 TCs) comparing citrate 5% to heparin (5000 U/mL) [13]. The rate of thrombus aspirated from the TC was doubled with citrate (14% versus 7%, *P* < 0.001); however, the rate of thrombolytic therapy was not significantly different (*P* = NS). In a larger (*n* = 232) recently published randomized controled trial comparing 46.7% citrate lock versus heparin lock (5,000 U/mL), the need for thrombolytic therapy was greater in the citrate group (8.2 versus 4.3/1000 days, *P* < 0.001) [17]. One advantage of citrate lock is that the bleeding event rate is reported to be significantly lower. In the first study, which included both tunneled (*n* = 98) and nontunneled (*n* = 193) HD catheters in acute and chronic renal failure patients, citrate lock (30%) was associated with a 70% reduction in major bleeding events when compared to heparin lock (5,000 U/mL), (*P* = 0.01) [15]. In the second study of 61 patients with HD TCs, there were significantly fewer systemic bleeding events using 4% citrate lock (7 in 32 patients) versus heparin lock (5,000 U/mL) (21 in 29 patients) (*P* = 0.035) [12].

Citrate and other chelating agents have been shown, in vitro, to inhibit biofilm formation and growth of *Staphylococcus aureus* and *Staphylococcus epidermidis* at concentrations greater than 0.5%. In contrast, heparin stimulated biofilm formation in this study [55]. Clinical trials using 4% citrate lock have not shown to lower TC-related bacteremia (CRB) rates, with the exception of one study [10]. In a prospective, nonrandomized trial, a significant reduction in CRB rates was observed when the TC locking protocol changed from a time period using heparin lock to a period of citrate lock 4% use; however, these findings are confounded by the concurrent initiation of a topical polyantibiotic ointment protocol, applied to the TC exit site, during the study period [10]. In two other studies comparing 4% citrate to heparin, there was no significant difference in CRB rates between locking agents [11, 12]. The ability of citrate to inhibit biofilm formation and bacterial growth is highly concentration dependent. In an in vivo study, Ash et al. observed a reduction in CRB when concentrations of 23% citrate or higher were used as a HD TC locking solution [56]. In a large randomized controlled trial, 30% citrate lock was associated with a significant reduction in the CRB rate (1.1 versus 4.1/1000 TC-days, *P* < 0.001) and fewer admissions for TC-related infections (0.7 versus 2.7 per 1000 TC days) [15]. More recently, a randomized controlled trial by Power et al. using 46.7% citrate lock versus heparin lock (5000 U/mL), failed to show a difference in CRB rates; however, the CRB rate was lower than that reported in other series (0.7/1000 TC days), which may have resulted in under-powering this study [17].

An additional potential advantage of 4% citrate lock is the estimated cost savings calculated in 2 Canadian studies. An 80–85% reduction in costs was calculated using citrate in comparison to heparin [10, 11]. In a 2008 position paper by the American Society of Diagnostic Interventional Nephrologists, the working group recommended that either heparin lock 1000 U/mL or 4% citrate lock be used in most TCs and that the injected volume not exceed the internal TC volume [57]. There are, however, advantages to citrate lock that make it a more desirable option, including lower bleeding risk, possible reduction in biofilm formation, avoidance of HAAb formation, lack of interference with prothrombin assays, and lower cost. At the present time, 4% citrate is used in the majority of Canadian HD units where it is available as a prefilled syringe (5 mL). (Citralok, MED-XL, Montreal, QC, Canada) In the United States 4% citrate is currently available in larger volume bags (250–500 mls) requiring preparation by the HD unit staff.

### 5.1. Novel Catheter Locking Solutions

Recently, Maki et al. reported the results of a multicentered, randomized, controlled trial using a novel catheter lock solution containing sodium citrate, methylene blue, methylparaben, and propylparaben (C-MB-P) in comparison to heparin catheter lock for HD TCs. The use of the C-MB-P solution was associated with a lower incidence of TC loss due to patency failure (0 versus 4, *P* = 0.04) and a lower rate of TC-related bacteremia (RR 0.29; CI 0.12–0.70; *P* = 0.005) [58].

## 6. Thrombolytic Agents as Catheter Locking Solutions

Fibrinolytic agents have been studied as an alternative to heparin for use as a TC locking solution. The potential advantage of these agents is the prevention of TC-related infections and improved TC patency. In a meta-analysis of 5 randomized controlled trials in 991 cancer patients using TCs for chemotherapy, urokinaselock or flush was associated with a significant reduction in TC-related infection (HR 0.77, 95% CI 0.60–0.98, *P* = 0.01) [59]. In vitro, alteplase has been shown to modestly inhibit *Staphylococcus aureus* biofilm formation in concentrations ≥0.5 mg/mL [55]. Prevention of HD TC thrombosis using a tissue plasminogen activator (TPA) interdialytic lock was first evaluated in 2 small clinical studies. In comparison to alteplase lock (1 mg/mL), heparin lock (5000 U/mL) was associated with more frequent thromboses (O.R. 2.4, 95% CI 1.5–4.0; *P* = 0.001). Schenk et al., using a prospective randomized crossover design, evaluated the efficacy of TPA lock versus heparin lock (1000 U/mL) in 12 HD TCs [18]. TPA lock was associated with significantly improved blood flow rates, lower venous pressures, and fewer complications. In contrast, TC thrombosis, requiring fibrinolytic intervention, occurred in 20% of patients during the heparin period. There was no difference in bleeding or infectious events between the groups. The “Pre-CLOT” (Prevention of Catheter Lumen Occlusion with r-TPA versus heparin) randomly assigned 225 patients with a newly inserted TC to heparin (5000 U per milliliter) three times per week or recombinant tissue plasminogen activator (rt-PA) (1 mg in each lumen) substituted for heparin at the midweek session (with heparin used in the other two sessions) [20]. TC malfunction occurred in 40 of the 115 patients assigned to heparin only (34.8%) and 22 of the 110 patients assigned to rt-PA (20.0%) (hazard ratio, 1.91; 95% confidence interval (CI), 1.13 to 3.22; *P* = 0.02). Catheter-related bacteremia occurred in 15 patients (13.0%) assigned to heparin only, as compared with 5 (4.5%) assigned to rt-PA (corresponding to 1.37 and 0.40 episodes per 1000 patient-days in the heparin and rt-PA groups, resp.; *P* = 0.02). The risk of bacteremia from any cause was higher in the heparin group than in the rt-PA group by a factor of 3 (hazard ratio, 3.30; 95% CI, 1.18 to 9.22; *P* = 0.02). The risk of adverse events, including bleeding, was similar in the two groups. The incremental cost of caring for patients with rt-PA as compared with heparin was $1,173 per patient (Canadian dollars).

## 7. Oral Agents for Prophylaxis to Inhibit Catheter Dysfunction

The use of warfarin alone or in combination with an antiplatelet agent has been evaluated for primary prevention of HD TC dysfunction in 3 randomized controlled trials. The first study was a randomized placebo controlled trial which included 85 HD patients receiving their first TC and compared fixed dose warfarin (1 mg/day) to placebo [23]. This minidose of warfarin was previously shown to be associated with a 75% reduction in TC thrombosis rates in cancer patients [60]. Unfortunately, minidose warfarin was not associated with improvement in primary unassisted patency or assisted TC survival in HD TCs. There was no increase in bleeding events with mini-dose coumadin. Another important finding of this study was that an INR of <1.00 was associated with significantly greater risk of TC loss due to dysfunction (HR = 4.0, 95% C.I. 1.1–14.5; *P* = 0.04) and earlier need for thrombolytic therapy (H.R 2.8, 95% CI 1.3–6.1, *P* = 0.009) [17]. The second study compared low-intensity monitored warfarin (target INR 1.5–1.9) to placebo in HD patients with newly placed TCs [24]. Warfarin was ineffective in preventing TC dysfunction. The third study included 144 newly inserted HD TCs, compared low-intensity warfarin (targeted to an INR of 1.8–2.5) and ticlodipine (250 mg/day) initiated within 12 hours after TC insertion (primary prevention) to a control group (ticlodipine alone) who received warfarin after the first thrombosis (secondary prevention) [27]. There was a significant reduction in TC thrombosis/dysfunction when the combined regimen of warfarin and ticlodipine was used as primary prevention compared to secondary prevention (0.16 versus 1.65 thrombotic events/patient year, *P* < 0.001), improvement in TC flow rates, and fewer TC removals for dysfunction (2.4% versus 17.5%, *P* < 0.001). It should be noted, however, that more patients in the primary prevention group achieved adequate anticoagulation than in the secondary prevention group (92% versus 65%, *P* < 0.05). There were no bleeding events associated with the warfarin/ticlodipine combination.

Warfarin use for secondary prevention was also evaluated by Zellweger et al. in a prospective study of 35 HD patients considered high risk for TC dysfunction given low-intensity warfarin (INR 1.5–2.0) compared to low-risk TC patients [25]. Therapeutic warfarin with adequate anticoagulation was associated with improved dysfunction-free TC survival at 9 months (47.1%) in comparison to that with inadequate anticoagulation (8.1%) (*P* = 0.01). In an observational study by Obialo et al., 63 HD patients with TCs who were already receiving chronic aspirin (*n* = 21, A, 325 mg/day) or therapeutic warfarin (*n* = 11, W, target INR 2-3) therapy for an underlying cardiovascular indication, and controls (*n* = 31, C) not taking either medication were prospectively monitored [26]. Both aspirin and warfarin were associated with improved primary TC patency at 120 days in comparison controls (C) (A 91%, W 73%, C 29% (*P* < 0.001)). Gastrointestinal bleeding rates were significantly higher in those patients on aspirin and warfarin in comparison to controls (A 24%, W 18%, C 0%, *P* < 0.02), and elderly patients were at highest risk of bleeding (H.R. 1.14, 95% C.I. 1.0–1.3, *P* = 0.008).

In summary, the combination of low-intensity warfarin, to achieve a target INR of 1.5–2.0, with or without ticlodipine, has not been consistently shown to be efficacious in maintaining HD TC patency, particularly when used as primary prevention. Although this therapeutic combination was not associated with an increased bleeding risk in the trial by Col*Ì* et al., low-intensity warfarin was associated with a significant bleeding risk in HD patients when used for the prevention of TC [24] or arteriovenous graft thrombosis [61]. In this study, by Crowther et al., warfarin was associated with 6 major bleeding events (all patients were also on aspirin), whereas none in the placebo group had major bleeds (*P* = 0.03). Furthermore, warfarin had no effect on arteriovenous graft survival. The use of warfarin should be reserved for high-risk patients with recurrent TC dysfunction, using a low-intensity protocol (target INR of 1.5–2.0).

## 8. Heparin-Coated Catheters

Heparin-coated HD TCs (HCC) have not been shown to reduce the need for thrombolytic therapy or improve TC survival in 2 retrospective trials [21, 22]. However, in one of the studies, Jain et al. reported a significantly lower TC-associated bacteremia rate in the HCC group (34% HCC versus 60% noncoated HD TCs, *P* < 0.001) [22]. In the second study, Clark et al. found no difference in infection between HCC and noncoated HD TCs; however, the mean observation period was relatively brief; (48–74 days) [21].

## 9. Conclusion

TCs remain uniquely vital to provide not only short-term renal replacement therapy but also long-term HD to thousands of patients every day. TC malfunction and infections are the two most important complications from TC and are responsible for higher morbidity and mortality in many dialysis patients. Recent studies have elucidated the importance of bacteria in the formation of biofilms and the development of fibrin sheaths that play a major role in the development of TC malfunction and TC-associated infections.

Most efforts to prevent dialysis TC malfunction have focused on the instillation of locking solutions in TCs. Heparin and citrate prevent clot formation in dialysis TCs and are the most commonly used TC lock solutions. Thrombolytic agents have been recently shown to be more effective in reducing TC-related infections and improving TC patency. Systemic administration of oral anticoagulation could be of benefit in selected patients at high risk for recurrent TC dysfunction, but is associated with greater bleeding risk. The role of antiplatelet agents for prevention of TC dysfunction has not been defined.

## 10. Future Directions

Important areas of future research include development of modified TCs with new designs and thrombosis-resistant properties and novel locking solutions and interventions to prevent or reduce biofilm and fibrin sheath formation. Furthermore, attaining a better understanding of the pathogenesis of biofilm and fibrin sheath development may allow for the development of novel therapies to manage these entities through translational research. Thus, other additional future areas of research could focus on (1) the molecular and genetic basis of biofilm and fibrin sheath development, (2) therapeutic agents that target the biofilm phenotype and community signaling-based agents that prevent the formation, or promote the detachment, of biofilms, and (3) whether a blood-based biomarker could be associated with the conversion from bacteria colonization into infection and fibrin sheath development [35, 47].

## Figures and Tables

**Table 1:** Studies evaluating therapies for the prevention of HD catheter dysfunction.

	Reference	Study design *N *	Treatment groups			Outcome	Effect	*P*
*Catheter locking agents*								

Heparin	Thomas, 2007 [7]	*P * *N* = 273	Heparin (1000 U/mL)		Heparin (10 000 U/mL)	Catheter dysfunction (per 1000 HD sessions)	Low H/high H 6.7 versus 7.6	NS
						Thrombolytic therapy (per 1000 HD session)	Low H/high H 26.6 versus 8.2	<0.001

Heparin	Holley, 2007 [8]	*R * *N* = 64	Heparin (1000 U/mL)		Heparin (10 000 U/mL)	Thrombolytic therapy (per 6 months)	Low H/high H 63% versus 31%	<0.001

Citrate (4%)	Buturovic, 1998 [9]	RCT *N* = 30	Citrate 4%	Heparin (1666 U/mL)	Polygeline (3.5%)	Catheter survival (days)	C/H/P 51/23/32	<0.01

Citrate (4%)	Lok, 2007 [10]	*P * *N* = 250	Citrate 4%		Heparin (5000 U/mL)	Thrombolytic rate (per 1000 days)	C/H 3.3 versus 5.5	<0.001
						Catheter removal for poor flow (per 1000 days)	C/H 1.65 versus 2.98	0.042

Citrate (4%)	Grudzinski, 2007 [11]	*R * *N* = 307	Citrate* 4%		Heparin** (10 000 U/mL)	Thrombolytic rate (per 1000 days)	C/H 3.23 versus 4.10	0.07
						Catheter removal for poor flow (per 1000 days)	C/H 1.88 versus 1.81	NS

Citrate (4%)	MacRae, 2008 [12]	RCT *N* = 61	Citrate 4%		Heparin (5000 U/mL)	Thrombolytic therapy (6 months)	C/H 41% versus 45%	NS

Citrate (5%)	Hendrickx, 2001 [13]	RCT *N* = 19	Citrate 5%		Heparin (5000 U/mL)	Thrombolytic therapy (per HD session)	C/H 8% versus 1%	NS
						Aspiration of thrombus (6 months)	C/H 14% versus 7%	<0.001

Citrate (30%)	Stas, 2001 [14]	*P * *N* = 11	Citrate 30%		Heparin (5000 U/mL)	Aspiration of thrombus	C=H	NS

Citrate (30%)	Weijmer, 2005 [15]	RCT *N* = 291	Citrate 30%		Heparin (5000 U/mL)	Thrombolytic Therapy (6 months)	C/H 47% versus 44%	NS
						Catheter removal for poor flow (per 1000 days)	C/H 3.2 versus 3.6	NS

Citrate (47%)	Bayes 1999 [16]	*P * *N* = 10	Citrate, 46.7%		Heparin (5000 U/mL)	Blood flow rate	C=H	NS

Citrate (47%)	Power, 2009 [17]	RCT *N* = 232	Citrate, 46.7%		Heparin (5000 U/mL)	Thrombolytic therapy (per 1000 days)	C/H 8.2 versus 4.3	<0.001

Tissue plasminogen activator	Schenk, 2000 [18]	*P * *N* = 12	r-TPA (1 mg/mL interdialytic lock)		Heparin (1000 U/mL)	Blood flow rate mL/min	TPA/H 237 versus 208	0.001
						Thrombolytic therapy (4 months)	TPA/H 0% versus 20%	—

Tissue plasminogen activator	Gittins, 2007 [19]	*P * *N* = 9	r-TPA (1 mg/mL interdialytic lock)		Heparin (1000 U/mL)	Aspiration of thrombus	H > rTPA: O.R. 2.4	0.001
						Clot volume	H > rTPA: O.R. = 1.9	<0.001

Tissue plasminogen activator	Hemmelgarn, 2011 [20]	RCT *N* = 225	r-TPA (1 mg/mL interdialytic lock midweek heparin 5000 U/Ml in other 2 sessions)		Heparin (5000 U/mL)	Catheter malfunction	rTPA < heparin HR 1.91	0.02

						Catheter-related bacteremia (episodes/1000 catheter days)	rTPA<Heparin 0.40 versus 1.37	0.02

*Modified catheters *								

Heparin- coated catheters	Clark, 2009 [21]	*R * *N* = 88	Heparin-coated catheter (+ heparin lock 5000 U/mL)		Noncoated catheter (+ heparin lock 5000 U/mL)	Primary patency (at 3 months)	HCC/NCC 82% versus 76%	NS
						Thrombosis rate (per 1000 days)	HCC/NCC 0.8 versus 0.4	NS

Heparin- coated catheters	Jain, 2009 [22]	*R * *N* = 175	Heparin-coated Catheter (+ Hepain lock 5000 U/mL)		Non-coated Catheter (+ Hepain lock 5000 U/mL)	Cumulative catheter survival (at 6 months)	HCC/NCC 48% versus 41%	NS
						Thrombolytic therapy (per 1000 days)	HCC/NCC 1.8 versus 1.8	NS

*Oral agents*								

Warfarin (mini dose)	Mokrzycki, 2001 [23]	RCT *N* = 85	Warfarin (1 mg)		Placebo	Primary catheter patency (at 1 year)	W/P 58% versus 48%	NS
						Assisted primary catheter patency (at 1 year)	W/P 20% versus, 18%	NS

Warfarin (low intensity)	Wilkieson, 2011 [24]	RCT *N* = 174	Warfarin (INR 1.5–1.9)		Placebo	Primary catheter patency	W/P 46% versus 47%	NS
						Catheter removal for dysfunction	W/P HR = 0.87	NS

Warfarin (low intensity)	Zellweger, 2005 [25]	*P * *N* = 65	Warfarin (INR 1.5–2.0) (high-risk pts)		Controls (low-risk pts)	Primary catheter patency (at 9 months)	Anticoagulation (adequate versus inadequate) 47% versus 8%	0.01

Warfarin (high intensity)	Obialo, 2003 [26]	*P * *N* = 63	Aspirin 325 mg/d	Warfarin (INR 2-3)	Control	Primary catheter patency (number of days)	A/W/C 114/111/68	<0.001
						Catheter survival (at 4 months)	A/W/C 91%/73%/29%	<0.001

Warfarin (medium intensity) and ticlodipine	Coli, 2006 [27]	RCT *N* = 144	Primary prevention Warfarin (INR 1.8–2.5) + ticlodipine 250 mg/day (1°W + T)		Secondary prevention (INR 1.8–2.5) + Ticlodipine 250 mg/day (2°W + T)	Catheter dysfunction (1 year)	1°W + T/2°W + T 12% versus 52%	<0.01
						Catheter dysfunction (events per pt/year)	PWT/RWT 0.16 versus 1.65	<0.001
